# Supplementary material for: Phase-to-pattern inverse design paradigm for fast realization of functional metasurfaces via transfer learning
Source: Nat Commun. 2021 May 20;12:2974. doi: 10.1038/s41467-021-23087-y (PMC8137937; doi:10.1038/s41467-021-23087-y)
Supplement: Supplementary file 1 — Supplementary Information [file 41467_2021_23087_MOESM1_ESM.pdf]

Supplementary Information for

Phase-to-Pattern Inverse Design Paradigm for Fast Realization of Functional Metasurfaces via Transfer Learning

Ruichao Zhu[1], Tianshuo Qiu[1], Jiafu Wang[*1], Sai Sui[*1], Chenglong Hao[2], Tonghao Liu[1], Yongfeng Li[1], Mingde Feng[1], Anxue Zhang[3], Cheng-Wei Qiu[*2], Shaobo Qu[*1]*

1 Department of Basic Sciences, Air Force Engineering University, Xi'an 710051, PR China.

2 Department of Electrical and Computer Engineering, Faculty of Engineering, National University of Singapore, 4 Engineering Drive 3, 117583, Singapore

3 School of Electronics and Information Engineering, Xi'an Jiaotong University, Xi'an 710049, PR China

E-mail: wangjiafu1981@126.com; suisai_mail@foxmail.com; qushaobo@126.com; chengwei.qiu@nus.edu.sg

Supplementary Note 1. Generalization of other materials

In order to demonstrate that the transfer learning network (TLN) is effective in more scenarios, we tested two other dielectric substrate materials, that is, FR4 (ε_r = 4.3 and tanδ = 0.025) and RogersTMM10i (ε_r = 9.8 and tanδ = 0.002), to train the TLN model. The reflection phase is greatly influenced by the type of material. Different datasets for different materials were collected to train the TLN model. **Supplementary Figure 1** illustrated the training process of different material datasets. According to the performances for different materials, the accuracy of training set is higher than 90% and the accuracy of test set is higher than 80%. This convincingly proves that the TLN model is effective and can be generalized to more materials.

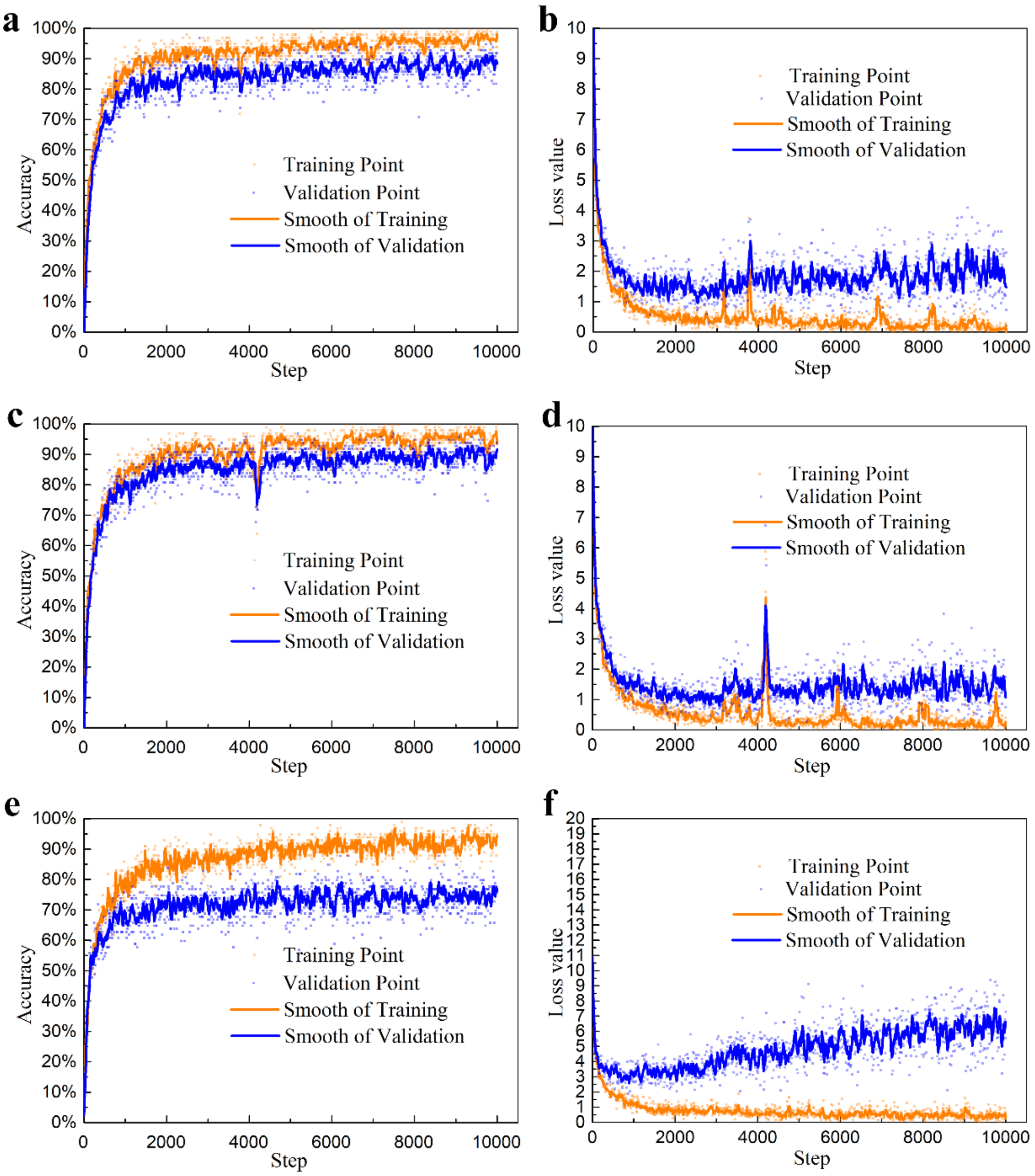

Supplementary Figure 1. The process of TLN training for different materials: (a) Accuracy of dataset for F4B material (b) Cross-entropy of dataset for F4B material (c) Accuracy of dataset for FR4 material (d) Cross-entropy of dataset for FR4 material (e)

Accuracy of dataset for RogersTMM10i material (f) Cross-entropy of dataset for RogersTMM10i material

Supplementary Note 2. Comparison of different neural networks

In order to demonstrate the effectiveness of TLN model, we fabricated different neural networks to test the training data. We employed some representative networks, i.e., Back-Propagation (BP) network, CNN network and Mobile network, to test the dataset.

In this section, we adopted the BP network and CNN network as below.

The BP network consists of 3 layers: input layer (meta-atom image), hidden layer (10 neurons) and output layer (360 categories). The BP network is shown in **Supplementary Figure 2(a)**.

The CNN network consists of 6 layers: input layer (meta-atom image), Convolution layer (3*3), Max Pooling layer (2*2), Convolution layer (3*3), Max Pooling layer (2*2) and Fully Connected Layer (360 categories). The CNN network is shown in **Supplementary Figure 2(b)**.

For further comparison, we chose Mobile Net for comparison experiment. Mobile Net is a lightweight network, only 16MB in size. This network is also trained using transfer learning. The Mobile Net is shown in **Supplementary Figure 2(c)**. [1]

In this work, we employed the Inception V3 to achieve the phase prediction of meta-atoms. The structure of Inception V3 is shown in **Supplementary Figure 2(d)**. [2]

In ImageNet, the performance of Mobile Net is TOP-1 = 0.704 and TOP-5 = 0.895, while the performance of Inception V3 is TOP-1 = 0.779 and TOP-5 = 0.937. Therefore, the performance of model in ImageNet will affect the performance of transfer learning to some extent. Although the accuracy is a little reduced when a lighter model is adopted, TLN model still achieve a better performance. Therefore, transfer learning is still an effective way of reducing training data and of improving the training performance.

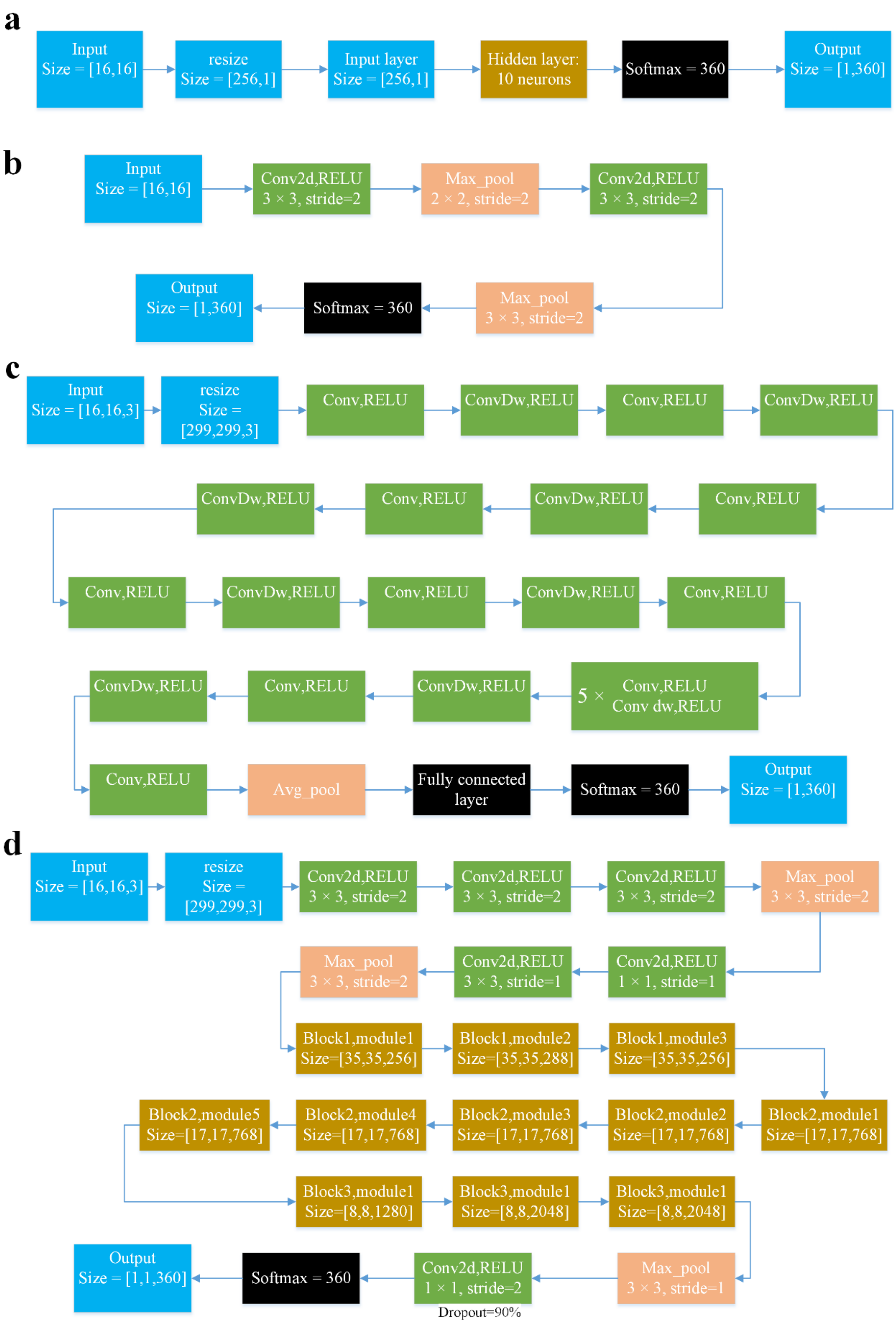

Supplementary Figure 2. Structures of different network: (a) BP network; (b) CNN network; (c)Mobile Net; (d) Inception V3

Supplementary Note 3. Training process of deep learning Inception V3

In order to explore the performance of deep learning, we provide the training process of deep learning Inception V3. From the **Supplementary Figure 3**, we can conclude that the performance of deep learning Inception V3 is poor. The accuracy of training data is around 30%, and the accuracy of validation is around 10%. The training data is overfitting.

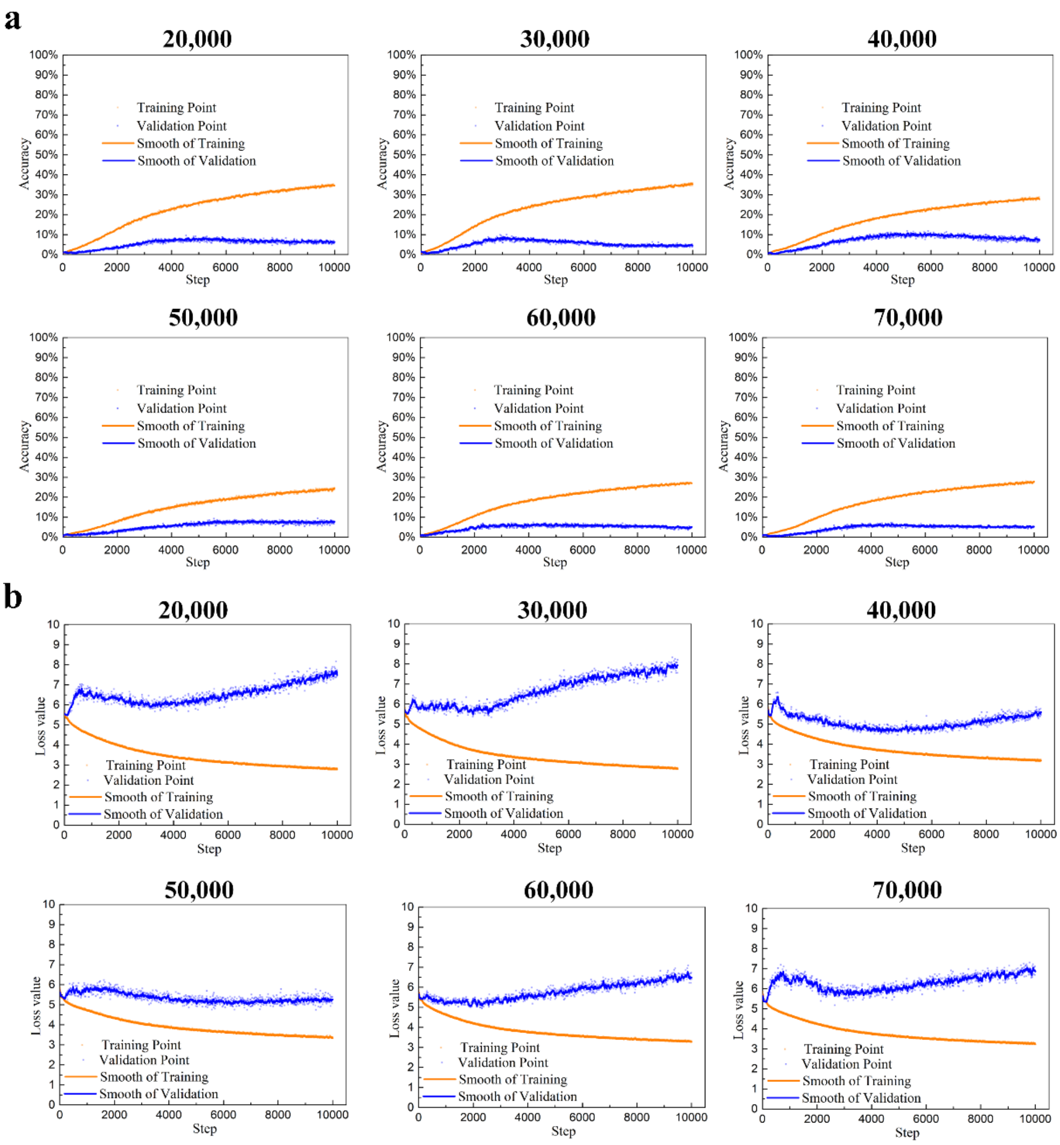

Supplementary Figure 3. The training process of deep learning Inception V3: (a) the variation of accuracy (b) the variation of loss

Supplementary Note 4. More validation

The TLN model can establish a holographic phase-pattern library of meta-atoms, with full phase span to achieve inverse design for fast realization of functional metasurfaces. In order to verify the validity and accuracy of our proposed method, we designed two multi-focus planar metasurfaces as validation.

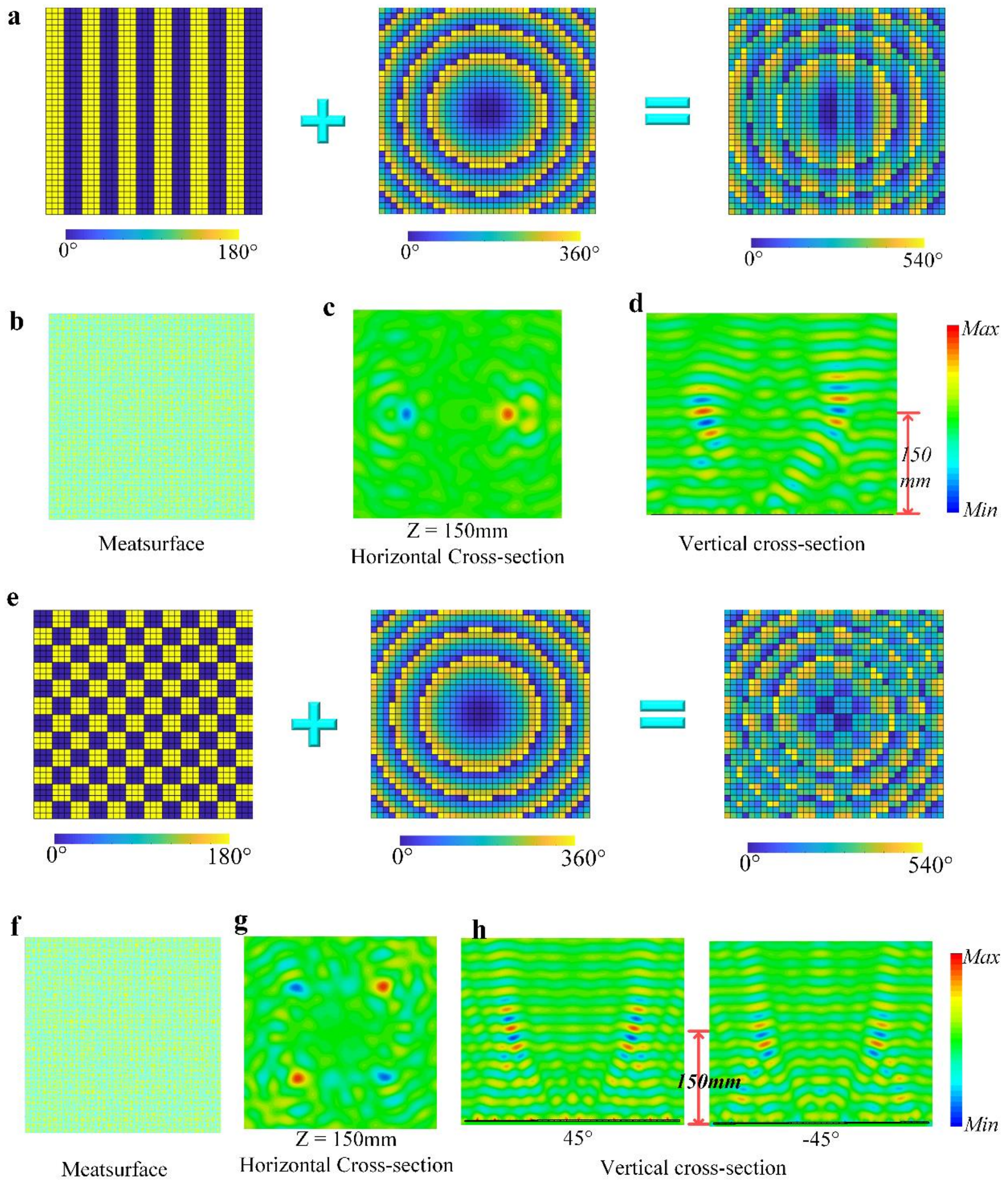

Supplementary Figure 4. Design of multi-foci planar metasurfaces: (a) Phase profile for double-foci focusing; (b) generated double-foci focusing metasurface; (c) fields on the plane Z = 150 mm for the double-foci focusing metasurface; (d) fields on X-O-Y plane for the double-foci focusing metasurface; (e) Phase profile for four-foci focusing; (f) generated four-foci focusing metasurface (g) fields on the plane Z = 150 mm for the four-foci focusing metasurface; (h) fields on ±45° vertical planes for the four-foci focusing metasurface;

From **Supplementary Figure 4**, we can conclude that the metasurface can focus reflected waves at multiple foci and all the focal lengths are 150 mm. The simulation results of double-foci focusing and four-foci focusing metasurfaces are in good accord

with theoretical design, which proves that our model can effectively implement various kinds of functional metasurface design, even with very complex functions.

Supplementary References

1. Howard, A. G. *et al.* MobileNets: Efficient Convolutional Neural Networks for Mobile Vision Applications. (2017). https://arxiv.org/abs/1704.04861
2. Szegedy, C., Vanhoucke, V., Ioffe, S., Shlens, J. & Wojna, Z. Rethinking the Inception Architecture for Computer Vision. *Proc. IEEE Comput. Soc. Conf. Comput. Vis. Pattern Recognit.* **2016-Decem**, 2818–2826 (2016). https://arxiv.org/abs/1512.00567
